# The epidemiology of laboratory-confirmed Hepatitis B Virus infection in the general population of South Africa, 2016-2018

**DOI:** 10.11604/pamj.2024.48.172.40907

**Published:** 2024-08-13

**Authors:** Mashudu Teresa Lamola, Alfred Musekiwa, Alex de Voux, Carl Reddy, Portia Chipo Mutevedzi

**Affiliations:** 1 School of Health Systems and Public Health, Faculty of Health Sciences, University of Pretoria, Pretoria 0002, South Africa,; 2 South African Field Epidemiology Training Program, National Institute for Communicable Diseases, National Health Laboratory Services, Johannesburg 2193, South Africa,; 3 Division of Epidemiology and Biostatistics, School of Public Health, University of Cape Town, Cape Town 7925, South Africa,; 4 Training Programs in Epidemiology and Public Health Interventions Network (TEPHINET), A program of the Task Force for Global Health, 325 Swanton Way, Decatur, GA 30030, USA,; 5 Emory Global Health Institute, Emory University, Atlanta, GA, USA

**Keywords:** Hepatitis B virus, Prevalence, South Africa

## Abstract

**Introduction:**

despite the introduction of the Hepatitis B Virus (HBV) vaccine in South Africa in 1995, HBV remains endemic. South Africa's HBV vaccine coverage for the third dose was 71% in 2015. Information on the HBV prevalence in South Africa in recent years is limited, therefore, we estimated HBV prevalence and described annual trends.

**Methods:**

we conducted a retrospective descriptive study of data extracted from the Notifiable Medical Conditions Surveillance System, and estimated HBV prevalence per 100,000 population using the mid-year population estimates obtained from Statistics South Africa, for the 2016-2018 period.

**Results:**

in total, 105 308 laboratory-confirmed HBV cases were analysed, of which 50.2% (53 895/105 308), 95% CI (49.9-50.5) were males. HBV prevalence for males was 34.1 in 2016, 84.1 in 2017, and 72.3 per 100,000 population in 2018. The age group with the highest HBV cases and prevalence were ages 15-49 years having 80.5% (n=84 718), with 52.2 in 2016, 123.3 in 2017, and 99.6 per 100 000 population in 2018. Between 2016 and 2018, South Africa had an overall HBV prevalence of 33.8, 82.6, and 68.8 per 100,000 population, respectively. KwaZulu-Natal province had the highest number of HBV cases with 37.8% (n=39 851) however, Mpumalanga province had the highest HBV prevalence with 73.2 in 2016, 188.8 in 2017, and 126.5 per 100,000 population in 2018.

**Conclusion:**

our results indicated a high HBV prevalence is reflective of the group prior to the HBV vaccine introduction in South Africa.

## Introduction

Hepatitis B virus (HBV) infection is an important public health problem globally. An estimate of approximately 257 million people have chronic HBV infection globally, approximately 60 million in Africa [1,2]. Although HBV infections occur all over the world, there is variation in the geographic distribution of HBV carriage. Sub-Saharan Africa (SSA) is categorised as a high-endemic area by the World Health Organization (WHO) [1,3]. The major mode of transmission of HBV is Mother-to-Child transmission (MTCT) in high endemic settings worldwide [4]. Vertical transmission of HBV to infants at birth or in early infancy causes an increased risk of chronic hepatitis [5-7]. Despite the effectiveness of HBV vaccination for newborns of infected mothers, in cases of a high viral load and Hepatitis B e antigen (HBeAg) positivity, there is a residual risk of HBV transmission to the newborn despite the introduction of prophylaxis [8].

Human immunodeficiency virus (HIV) and HBV are both of public health concern, have similar routes of transmission, and have no curative treatment to date with HBV being 100 times more infectious than HIV [3,5,6,9,10]. However oral antiviral systematic treatment can be used as potent drugs to suppress HBV, slow down the progression of cirrhosis, reduce the incidence of liver cancer, and improve long-term survival [9]. The global prevalence of HBV infection among HIV-infected persons was 7.4% (2.7 million) in 2022 [11]. Given the elevated prevalence of HBV among HIV-infected individuals (HBV/HIV-coinfected), in 2015, WHO recommended antiretroviral therapy (ART) initiation for all HIV-positive patients diagnosed with chronic liver disease, regardless of the CD4 count [12,13]. Among HIV-infected individuals, it has been shown that chronic HBV advances faster to cirrhosis, hepatocellular carcinoma (HCC), and end-stage liver disease, as compared to individuals only infected with HBV [13-15]. There are effective ARTs against both HIV and HBV, and simplified treatment algorithms to treat HBV/HIV coinfections [16]. In a country like South Africa where HIV prevalence is high, coupled with significant HBV prevalence, surveillance of both HBV and HIV remains crucial [17-19].

In 1995, South Africa introduced and added the HBV vaccine to the national schedule of the Expanded Programme on Immunization (EPI-South Africa) to be administered at 6, 10, 14 weeks, and 18 months [20-23]. The vaccine coverage varies from province to province, however, in South Africa, the HBV vaccine coverage with three doses averaged 85.9%, ranging from 82.3% (2012) to 89.8% (2017) [24,25]. Despite South Africa being a high-endemic HBV area and HBV being a category 2 notifiable medical condition (NMC), there is currently limited data on the prevalence of HBV. To address this limitation, we conducted a retrospective descriptive study, to estimate HBV prevalence among the general population of South Africa from January 2016 to December 2018.

## Methods

**Study design and study setting:** we performed a retrospective descriptive analysis of all laboratory-confirmed HBV cases reported to the national NMC surveillance system from January 2016 to December 2018. The NMC surveillance system is housed at the National Health Laboratory Services (NHLS) and collects data from both public and private health sectors in South Africa. HBV cases were reported from all nine provinces of South Africa; Eastern Cape, Free State, Gauteng, KwaZulu-Natal, Limpopo, Mpumalanga, North West, Northern Cape, and Western Cape.

**Laboratory testing:** active HBV positivity was determined using Polymerase Chain Reaction (PCR) on blood specimens whilst serology was used to detect antibodies against HBV. Acute hepatitis B is diagnosed in individuals who are positive for HBsAg and have indications of disturbed liver function, symptoms such as lethargy, nausea, fever, anorexia for a few days followed by jaundice, pale stools, and dark urine, and a risk history suggesting recent infection. The HBV incubation period is approximately 45-180 days. A chronic hepatitis B infection is diagnosed when an individual repeatedly tests HBsAg-positive over 6 months, in the absence of acute symptoms or a risk history to suggest a recent infection. Chronic HBV infection can be lifelong.

**Case definitions:** an acute case of HBV was defined as a laboratory-confirmed diagnosis, i.e., IgM anti-HBc positive, or IgM anti-HBc +ve and HBsAg positive. A chronic case of HBV was defined as laboratory-confirmed HBsAg positive or dually positive for total anti-HBc and HBsAg.

**Data collection process:** for this study, we included all laboratory-confirmed HBV cases notified by HCPs and those sent automatically to the NMC database system by the LIS and received at the NHLS. The NHLS services over 80% of the South African population across all nine provinces with the remainder being serviced by private laboratories [26].

**Data analysis**

Data were retrieved and stored using Microsoft Excel 2016, cleaned by removing duplicates or inconsistencies, and analyzed using both STATA 15 (Stata Corp®, College Station, Texas, USA) and Excel 2016. All repeated tests of the same case were excluded to ensure that each case appeared once only and thereby avoid case duplication (by name, surname, date of birth, date of notification, age, and sex). Descriptive statistical analysis for measures of frequency by count and percent, was applied, with 95% Confidence Intervals [95% CI] included. Demographic factors of HBV cases were described by age, sex, and province. HBV prevalence was estimated per 100,000 population, using mid-year population estimates obtained from Statistics South Africa (Stats SA) from 2016 to 2018 as the denominators. Age was categorized as < 1, 1-4, 5-14, 15-49, 50-64, and =65 years. Sex-specific and age-specific prevalence estimates were calculated using sex-specific and age-specific mid-year population statistics for the three years as the denominators. The number of cases per month during 2016-2018, were displayed as a trend line graph, by province and age group, to describe the annual trends and seasonal variation of HBV cases. We also examined the proportions of HBV cases for each age group by sex between 2016 and 2018.

**Ethical statement:** this study obtained an ethical clearance certificate from the University of Pretoria (UP) ethics reference No.: 181/2019 and internal data allocation was done with the consent of the Head of the NMC, Division of Public Health Surveillance and Response of the National Institute for Communicable Diseases, Johannesburg, South Africa.

## Results

### Characteristics of HBV cases

**Figure 1:**
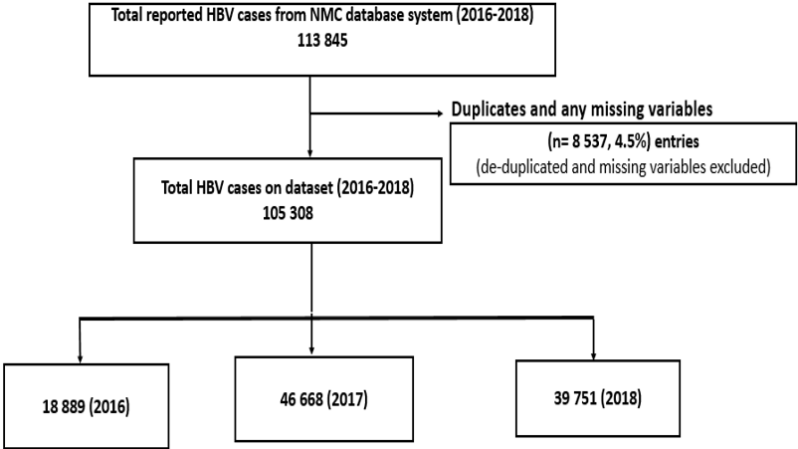
the eligibility criteria for laboratory-confirmed Hepatitis B Virus cases in the general population of South Africa, 2016-2018

A total of 4.5% (8 537/113 845) of records were excluded after de-duplicating and excluding records with missing information. A total of 105,308 HBV laboratory-confirmed cases reported to the NMC surveillance system from January 2016 to December 2018 were identified in the final dataset (Figure 1). There were 18 889 HBV cases in 2016, 46 668 in 2017, and 39 751 in 2018. Of the 105 308 HBV cases, the majority of cases, 50.2% (n=52 895), were among males and 80.5% (n=84 718) were 15-49 years old. The province with the highest proportion of HBV cases was KwaZulu-Natal constituting 37.8% (n=39 851), followed by Mpumalanga with 16.4% (n=17 282) while the lowest proportion of HBV cases were in the North West at 1.4% (n=1 465) (Table 1).

**Table 1:** demographic characteristics of laboratory-confirmed Hepatitis B Virus cases in the general population of South Africa, 2016-2018 (N=105 308)

Sociodemographic characteristics	HBV cases n (%)	95% CI
**Sex**		
Female	52 413 (49.8)	(49.4-50.0)
Male	52 895 (50.2)	(49.9-50.5)
**Age category (in years)**		
<1	679 (0.6)	(0.59-0.69)
1-4	136 (0.1)	(0.10-0.15)
5-14	489 (0.5)	(0.42-0.51)
15-49	84 718 (80.5)	(80.0-80.6)
50-64	15 089 (14.3)	(14.1-14.5)
≥ 65	4 197 (4.0)	(3.8-4.1)
**Province**		
Eastern Cape	14 464 (13.7)	(13.5-13.9)
Free State	1 838 (1.8)	(1.6-1.9)
Gauteng	14 686 (14.0)	(13.7-14.2)
KwaZulu-Natal	39 851 (37.8)	(37.5-38.1)
Limpopo	4 918 (4.7)	(4.5-4.8)
Mpumalanga	17 282 (16.4)	(16.1-16.6)
North West	1 465 (1.4)	(1.3-1.6)
Northern Cape	1 665 (1.6)	(1.5-1.7)
Western Cape	9 139 (8.7)	(8.5-8.8)

**N** = total number of HBV cases, **n** = Sample number of HBV cases, **n/N (%)** = proportion of HBV cases, **95% CI**= 95% Confidence Interval

### HBV prevalence estimates

**Figure 2:**
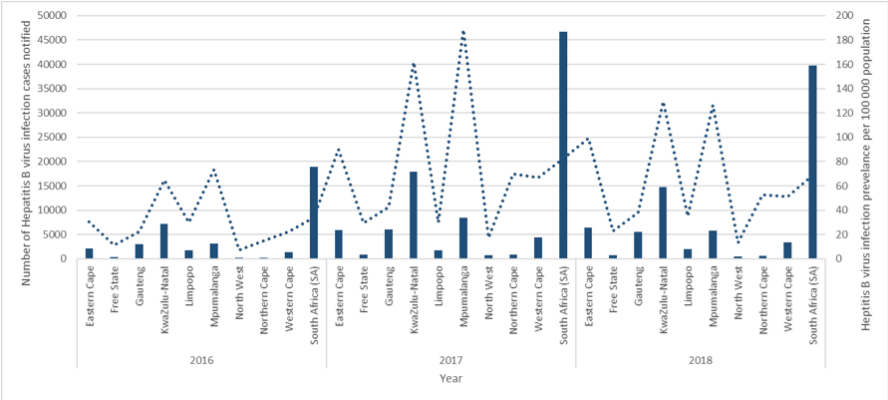
prevalence of Hepatitis B virus infection among the general population of South Africa, January 2016 to December 2018

The HBV prevalence in South Africa was 33.8 in 2016, 82.6 in 2017, and 68.9 in 2018, per 100,000 population. HBV prevalence was the highest in Mpumalanga with 73.2 in 2016, 188.8 in 2017, and KwaZulu-Natal with 129.5 per 100,000 population in 2018. The lowest HBV prevalence province was North West having 7.3 in 2016, 17.1 in 2017, and 13.3 per 100,000 population in 2018. The provinces that experienced an increase in HBV prevalence per 100,000 population throughout the three years included the Eastern Cape with 30.6, 89.9, and 99.0, for 2016, 2017, and 2018, respectively, Mpumalanga with 73.2, 188.8, and 126.5 for 2016, 2017 and 2018 respectively, and Limpopo having 29.8 in 2016, 29.9 in 2017 and 34.8 in 2018 (Figure 2).

**Figure 3:**
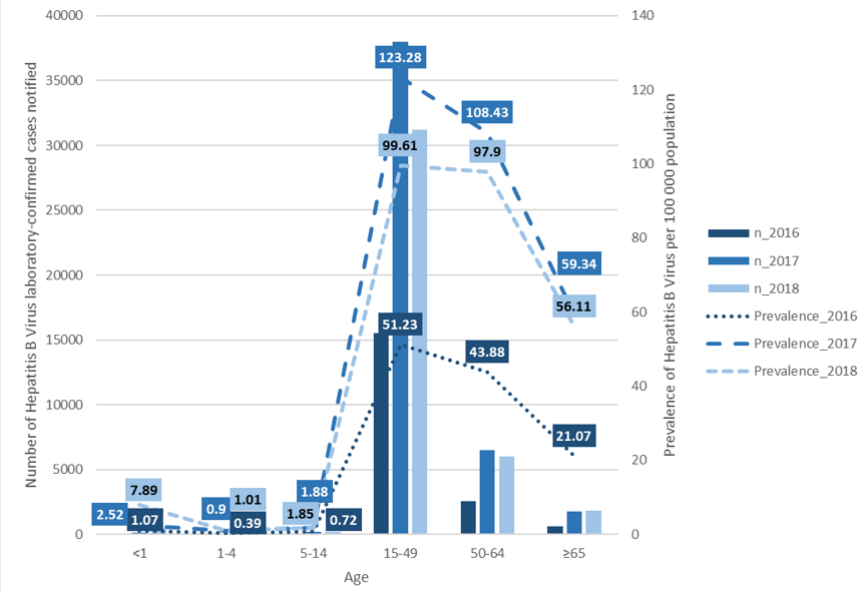
age-specific prevalence and number of cases of Hepatitis B virus infection among the general population of South Africa, January 2016 to December 2018

**Figure 4:**
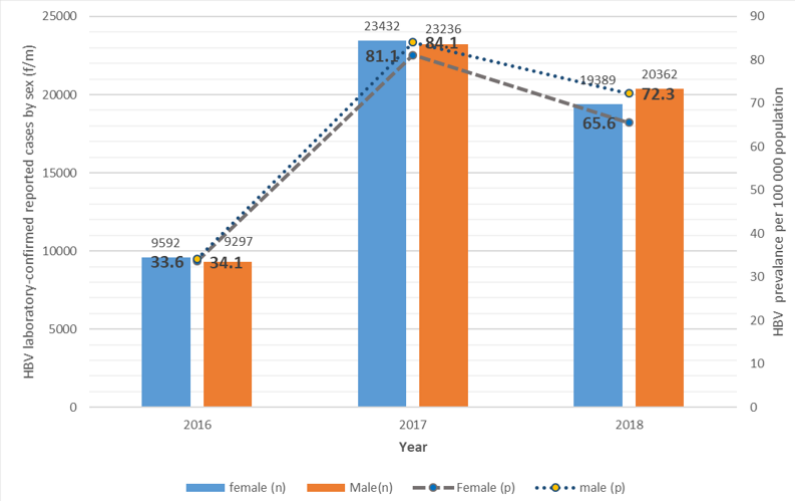
the sex-specific prevalence and number of cases of Hepatitis B virus infection among the general population of South Africa, January 2016 to December 2018

HBV prevalence was highest among those aged 15-49 years with 52.2 in 2016, 123.3 in 2017, and 99.6 in 2018, per 100,000 population. The lowest HBV prevalence was among those aged 1-4 years with 0.4 in 2016, 0.9 in 2017, and 1.0 in 2018, per 100,000 population, followed by ages 5-14 years having 0.7 in 2016, 1.9 in 2017, and 1.9 in 2018, per 100,000 population. Infants < 1 year had a general increase in HBV prevalence throughout the three years with 1.1, 2.5, and 7.9 respectively, per 100,000 population (Figure 3). The sex-specific HBV prevalence estimates show that males had a generally high prevalence as compared to females having 34.1 in 2016, 84.1 in 2017, and 72.3 in 2018, per 100 000 population. However, females had the majority of HBV cases in 2016 with 9 592 cases, and 23 432 cases in 2017 while males had the majority of HBV cases in 2018 with 20 362 cases (Figure 4).

**Figure 5:**
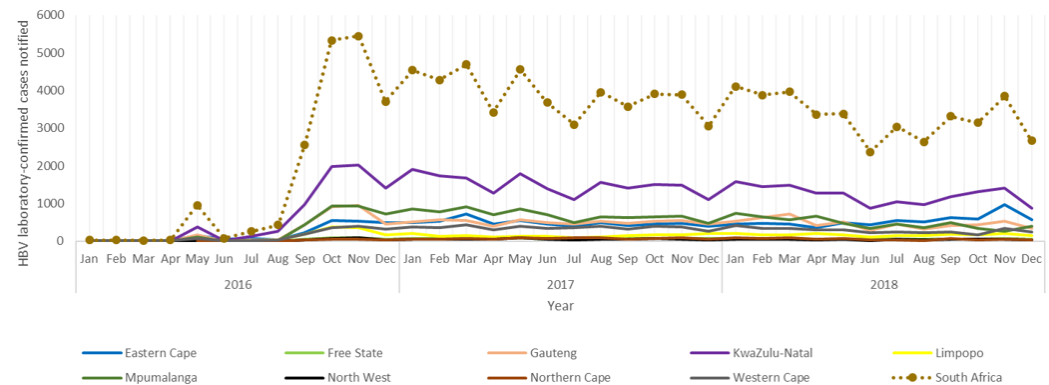
the annual distribution and trends of HBV laboratory-confirmed notified cases by months per province, in South Africa (2016-2018)

There were no specific trends or seasonality for the laboratory-confirmed HBV cases among the provinces of South Africa throughout the three years with the number of HBV cases fluctuating by year and month (Figure 5). However, there was a consistent drop in HBV notifications during December throughout the three years. South Africa had a generally rapid increase in HBV case notifications between August 2016 and October 2016. The steady increase in HBV cases was distributed as follows; 435 cases during August, 2 566 in September, and 5 331 in October.

**Figure 6:**
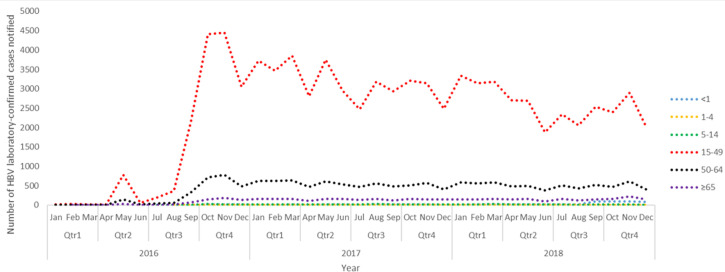
the annual distribution and trends of HBV laboratory-confirmed notified cases by quarter and months per age group, in South Africa (2016-2018)

Even though there were no specific trends or seasonality in the notified HBV cases among the age groups throughout the three years, there is evidence of a general decline in HBV cases nationally (Figure 6). A significant increase in HBV cases during 2016 (between August and October) was observed among ages 15-49 years and 50-64 years, followed by a significant decrease (between November and December 2016) for ages 15-49 years. The general trend illustrates an overall decline in the actual HBV cases in South Africa across all age groups.

Despite having a higher proportion of females compared to males per the sex-population ratio, males were mostly affected by HBV throughout the three years (Table 2). The provinces with higher HBV prevalence among females were Limpopo, Mpumalanga, and Northern Cape while KwaZulu-Natal, Gauteng, and Free State had a higher HBV prevalence in males throughout the three years. Although the overall HBV prevalence declined significantly from 2017 to 2018 across South Africa, Eastern Cape and Limpopo had an increase in both males and females. HBV prevalence in Eastern Cape province in 2016 were as follows (per 100 000 population): (females=31.8; males=29.2), in 2017 (females=90.9; males=88.9), and in 2018 (females=98.2; males=102.3). The HBV prevalence in Limpopo were as follows (per 100,000 population): in 2016 (females=22.2; males=18.0), in 2017 (females=32.4; males=27.1), and 2018 (females=35.6; males=33.8).

**Table 2:** the distribution and prevalence (per 100,000 population) of HBV laboratory-confirmed cases by sex per province per epidemiological year in South Africa, 2016 to 2018

Epidemiological Year												
	**2016**				**2017**				**2018**			
**Province**	**Female**		**Male**		**Female**		**Male**		**Female**		**Male**	
	**n (%)**	**Prevalence /100,000**	**n (%)**	**Prevalence /100,000**	**n (%)**	**Prevalence /100,000**	**n (%)**	**Prevalence /100,000**	**n (%)**	**Prevalence /100,000**	**n (%)**	**Prevalence /100,000**
**Eastern Cape**	1 170(54.2)	31.8	988(45.8)	29.2	3 117(53.3)	90.9	2 729(46.7)	88.9	3 320 (51.4)	98.2	3 140(48.6)	102.3
**Free State**	159 (49.7)	10.8	161(50.3)	11.6	366 (43.1)	24.8	483 (56.9)	34.7	277 (41.4)	18.2	392 (58.6)	27.3
**Gauteng**	1 487(49.1)	22.2	1 539(50.9)	22.6	2 941(48.4)	41.6	3 135(51.6)	43.5	2 714 (48.6)	37.3	2 870(51.4)	38.6
**KwaZulu-Natal**	3 388(47.1)	58.7	3 799(52.9)	71.6	8 452(47.2)	146.1	9 474(52.8)	179.2	6 702 (45.5)	41.6	8 036(54.5)	148.1
**Limpopo**	678 (57.8)	22.2	495(42.2)	18.0	989 (57.2)	32.4	740 (42.8)	27.1	1 089 (54.0)	35.6	927 (46.0)	33.8
**Mpumalanga**	1 764(55.7)	80.3	1 404(44.3)	65.9	4 569(54.5)	201.9	3 822(44.5)	175.2	3 011 (52.6)	130.5	2 712(47.4)	122.4
**North West**	144(52.2)	7.7	132(47.8)	6.9	321 (48.6)	16.8	339 (51.4)	17.4	242 (45.8)	12.1	287 (54.2)	14.5
**Northern Cape**	92 (53.2)	15.4	81 (46.8)	13.6	453 (53.6)	74.1	392 (46.4)	65.0	355 (54.9)	57.4	292 (45.1)	48.1
**Western Cape**	710(50.4)	22.5	698(49.6)	22.5	2 224(51.2)	67.5	2 122(48.8)	66.0	1 679 (49.6)	50.0	1 706(50.4)	52.3

## Discussion

Despite the prevention of HBV infection being a global public health priority, HBV remains a major public health challenge [27]. The association between HBV and HIV makes HBV control important in South Africa with significantly high HIV transmission rates. The World Health Organization (WHO) set goals to eliminate HBV. The target aims at a 90% reduction of chronic HBV incidence and a 65% reduction in deaths, and prevention of more than 7 million related deaths by 2030, worldwide [27,28]. The broad implementation of infant immunization against HBV infection, prevention and control programs, and blood safety practices have greatly reduced the burden of HBV between the years 2000 to 2010 [27,28]. Priority was based especially on groups at major risk of becoming chronic carriers. The priorities included the implementation of HBV vaccine-based strategies to prevent perinatal transmission, safe injection practices, and Hepatitis C Virus (HCV) treatment for persons who inject drugs, testing, and treatment for HBV-infected individuals [28]. However, this study does not indicate the groups at major risk (for instance, those who inject illicit recreational drugs, unhygienic tattooing practices, at risk of nosocomial infection, or occupational risks) due to the nature of the data collected into the NMCSS. The study described the characteristics of HBV cases and estimated HBV prevalence in the general population of South Africa. The strength of this study included the large sample size, detailed demographic data, rigorous laboratory tests for ascertainment of positive cases, and representativeness of outcomes in routine practice within South Africa as the NHLS covers over 80% of the public population.

Our study found that males represented a larger proportion of HBV cases, thus supported by the previous studies demonstrating the existence of sex differences in the development of HBV and HBV-related hepatocellular carcinoma (HCC) [29-31]. Similarly, a study in Zimbabwe indicated a significantly higher HBV prevalence for HBsAg positive males as compared to females [32]. Factors that have been shown to increase the likelihood of males being HBV infected included the use of substances such as injectable drugs. Additionally, the reuse, sharing, and use of contaminated injecting equipment, and living on the streets, were found to increase the likelihood of males being at higher risk of HBV infection as compared to females in the three cities of South Africa (namely, Cape Town, Durban, and Pretoria) [33]. Female HBV carriers are known to have generally lower viral loads than male carriers [34].

South Africa introduced the HBV vaccine in 1995, which marked 24 years post-vaccine introduction (as of 2019), and therefore individuals aged above 24 years are likely to experience high HBV prevalence. In our study, the most affected age groups were the 15-49 years followed by 50-64 years, and lastly ≥ 65 years. The majority of HBV cases in ages 15-49 years were among females at 50.4% while males had a slightly lower prevalence of 49.6%, mostly due to reasons that chronic HBV appears to progress more rapidly in males than in females, whilst HBV-related HCC tends to occur in men and postmenopausal women [30]. Approximately one-fifth of South African women in their reproductive ages 15-49 years are HIV positive, thus evidenced by the high proportions of HBV cases among females aged 15-49 years as compared to males of the same cohort [35]. During adolescence and early adulthood, sexual transmission becomes the dominant route of HIV and HBV, therefore, these might have influenced the high proportion of HBV cases among ages 15-49 years [36].

South Africa is among the high HBV prevalence areas of the sub-Saharan Africa (SSA) region constituting more than 8% of the population being infected [37]. Our study found that infants < 1 year, children 1-4 years, and older children and young adolescents of 5-14 years had general HBV proportions of < 1 and, however, there was an increase in the prevalence throughout the three years amongst these age groups. Since 1995, South African studies on children have found that the prevalence of chronic HBsAg carriage has decreased, commonly owing to the introduction of the HBV vaccine in 1995 [36]. The study however does not rule out the burden of horizontal transmissions as evidenced by the lowest HBV prevalence in infants < 1 year and children in general.

HIV/HBV coinfection has a significant impact on the natural history of HBV infection [13]. Approximately 2.7 million people living with HBV infection globally are also HIV infected, with an estimated 7.4% HIV/HBV infection prevalence [38]. South Africa has the largest HIV epidemic in the world, and high HBV prevalence is associated with HIV infection. South Africa introduced the Universal Test and Treat (UTT) programme on 1^st^ September 2016, making ART available to all HIV- infected persons regardless of CD4 count, together with same-day initiation (SDI) that advocates for ART initiation on the day of a patient´s HIV diagnosis [39]. The prevalence of chronic HBV infection in HIV-infected individuals is approximately 5%-15%, and HIV/HBV co-infected individuals have a higher level of HBV replication, with higher rates of chronicity, reactivation, occult infection, and HCC than individuals with HBV only [40].

During the study period in 2019, the estimated South African overall HIV prevalence rate in 2017 was approximately 12.6%, and 13.1% in 2018. The overall HIV prevalence among adults aged 15-49 years in South Africa was 20.6%, while 26.3% was among females and 14.8% was among males [41]. This study highlights that Mpumalanga and KwaZulu-Natal were the highest HBV prevalence provinces throughout the three years. The high HBV prevalence in KwaZulu-Natal might be influenced by the high HIV prevalence in this province, however, this is not the case in Mpumalanga [42]. The observed HBV prevalence by sex per province in South Africa indicates a continuous emphasis on higher HBV disease burden among males as compared to females. Similarly, a study done in Nigeria reported a higher HBV prevalence in males than females [43]. The higher HBV prevalence among males is thought to be related to the higher clearance rate of these viruses by females compared with males [44,45].

The rise in HBV notifications from 2016 to 2017 might be influenced by the re-engineered NMC system towards the improvement of notifications from the laboratory section. The re-engineered NMC surveillance system was characterized by the move from the manual to an automated real-time electronic reporting and feedback platform, real-time notification at the point of diagnosis via the NMC App with laboratories, private hospitals, and medical schemes data being automated in real-time to the NMC database. In 2017, HBV prevalence increased among males and females generally. The increase might be influenced by the improved notification of HBV cases nationwide. South Africa had a generally rapid increase in HBV case notifications between August 2016 and October 2016. As other provinces came onboard on the Trackcare system of the NHLS, this might have influenced the overall rapid increase in HBV notifications between August 2016 and October 2016.

The high prevalence of HBV in South Africa costs an estimated ZAR3.78 billion per five-year Action Plan on raising awareness among the health workforce and general population, which includes the information campaigns and training of health workers, strengthening knowledge of disease burden in surveillance, surveys, and special studies [46]. Additionally, high HBV prevalence increases the costs of prevention of Viral Hepatitis such as protection of HCPs, HBV vaccine birth dose, and prevention of mother-to-child transmissions (PMTCT), HBV testing, care, and treatment during screening, diagnosis, linkage to care, and drug therapy for HBV, management, and coordination of HBV program management, monitoring and evaluation, and policy development [46].

## Limitations of this study

The nature of the dataset did not allow an analysis of the burden of HBV/HIV coinfection because the HIV status is not captured in the NMC system and HIV is not notifiable. The nature of the dataset limited the study in the exploration of more demographic information such as race. The limited variables are reported at the time of notification hence we could not explore the association of HBV with a wide range of variables.

## Conclusion

Despite females being more than males nationwide as per sex-ratio statistics description, our study showed a generally high HBV case proportion and prevalence among males in the South African provinces throughout the three years of study. There was no specific trend or seasonality of the notified HBV laboratory-confirmed cases among provinces and age groups in South Africa throughout the three years, with the number of HBV cases fluctuating by year and month. An increase in HBV prevalence in the South African population of <15 years warrants and stimulates further monitoring nationwide. Several recommendations were given and our study concluded that male HBV programmes are needed to tackle the high HBV prevalence nationwide. This calls for HBV-vaccine catch-up programmes for high-risk groups (such as adults born before the HBV vaccine implementation and those that are HIV-infected, and even booster vaccines for those who have been vaccinated as evidenced by high HBV prevalence among ages 15-49 years). Additionally, we recommend public health interventions and policy improvement whereby HBV surveillance should be established as a provincial base as this will enable South Africa to identify improvements in the high HBV prevalence provinces. This will enable provinces to estimate the burden of HBV and identify populations at risk at a provincial level (even by district, and even sub-district), have timeously detection and response to the increase in HBV cases to prevent outbreaks and monitor place, person, and time trends and having a baseline history of a provincial HBV disease burden. Furthermore, we recommend continuous monitoring of HBV case trends and monitoring of HBV prevalence across the provinces of South Africa. Lastly, due to the 4.5% of missing variables analysed, we recommend training of HCPs and data capturers on the importance of data quality when notifying HBV cases.

### What is known about this topic

- *Hepatitis B virus (HBV) is a global public health threat and remains a major global health problem;*
- *HBV infection is endemic throughout sub-Saharan Africa, including South Africa;*
- *HBV remains a significant burden on public health in South Africa despite the introduction of HBV vaccines in 1995.*

### What this study adds

- *Males had a generally higher prevalence of HBV during the 2016-2018 period;*
- *The reproductive ages between 15-49 years contribute to 80.5% of HBV cases with HBV prevalence of 52.2 in 2016, 123.3 in 2017, and 99.6 in 2018, per 100 000 population;*
- *Despite KwaZulu-Natal having the highest HBV cases (37.5%), Mpumalanga province had the highest HBV prevalence throughout the three years.*

## References

[1] World Health Organization (WHO) (2017). Global hepatitis report 2017. World Health Organization.

[2] Nayagam S, Thursz M, Sicuri E, Conteh L, Wiktor S, Low-Beer D (2016). Requirements for global elimination of hepatitis B: a modelling study. The Lancet Infectious diseases.

[3] Nelson NP, Easterbrook PJ, McMahon BJ (2016). Epidemiology of Hepatitis B Virus Infection and Impact of Vaccination on Disease. Clinics in liver disease.

[4] Celen MK, Mert D, Ay M, Dal T, Kaya S, Yildirim N (2013). Efficacy and safety of tenofovir disoproxil fumarate in pregnancy for the prevention of vertical transmission of HBV infection. World Journal of Gastroenterology. WJG.

[5] Shimakawa Y, Seck A, Nayagam S, Toure-Kane C, Lemoine M (2018). Screening strategies to prevent mother-to-child transmission of hepatitis B in sub-Saharan Africa. The lancet Gastroenterology & hepatology.

[6] Idilman R (2017). Management of special patient groups with hepatitis B virus infection: The EASL 2017 Clinical Practice Guidelines. The Turkish journal of gastroenterology: the official journal of Turkish Society of Gastroenterology.

[7] Adekanle O, Ndububa DA, Olowookere SA, Ijarotimi O, Ijadunola KT (2015: 2015). Knowledge of hepatitis B virus infection, immunization with hepatitis B vaccine, risk perception, and challenges to control hepatitis among hospital workers in a Nigerian tertiary hospital. Hepatitis research and treatment.

[8] Gentile I, Borgia G (2014). Vertical transmission of hepatitis B virus: challenges and solutions. International journal of women's health.

[9] Farid Y, Martin C, Delforge M, De Wit S (2019). Epidemiology and clinical management of HIV-HBV coinfected patients in a large AIDS Reference Center in Belgium. Acta clinica Belgica.

[10] Spearman C, Sonderup MW, Botha J, Van der Merwe SW, Song E, Kassianides C (2013). South African guideline for the management of chronic hepatitis B: 2013. South African Medical Journal.

[11] World Health Organization (WHO) (2022). Hepatitis B key facts.

[12] Amini A, Varsaneux O, Kelly H, Tang W, Chen W, Boeras DI (2017). Diagnostic accuracy of tests to detect hepatitis B surface antigen: a systematic review of the literature and meta-analysis. BMC infectious diseases.

[13] World Health Organization (WHO) (2015). Guidelines for the Prevention Care and Treatment of Persons with Chronic Hepatitis B Infection: Mar-15. World Health Organization.

[14] Centers for Disease Control and Prevention Coinfection with HIV and Viral Hepatitis.

[15] HIV and Opportunistic Infections, Coinfections, and Conditions

[16] Dharel N, Sterling RK (2014). Hepatitis B Virus-HIV Coinfection: Forgotten but Not Gone. Gastroenterology & hepatology.

[17] Zuma K, Simbayi L, Zungu N, Moyo S, Marinda E, Jooste S (2022). The HIV Epidemic in South Africa: Key Findings from 2017 National Population-Based Survey. International journal of environmental research and public health.

[18] Pillay T, Cornell M, Fox MP, Euvrard J, Fatti G, Technau KG (2020). Recording of HIV viral loads and viral suppression in South African patients receiving antiretroviral treatment: a multicentre cohort study. Antiviral therapy.

[19] Meyer-Rath G, Johnson LF, Pillay Y, Blecher M, Brennan AT, Long L (2017). Changing the South African national antiretroviral therapy guidelines: The role of cost modelling. PloS one.

[20] Amponsah-Dacosta E, Lebelo RL, Rakgole JN, Selabe SG, Gededzha MP, Mayaphi SH (2015). Hepatitis B virus infection in post-vaccination South Africa: occult HBV infection and circulating surface gene variants. J Clin virol.

[21] Maja P, Dlamini N (2016). The Expanded Programme on Immunisation in South Africa: a story yet to be told: in practice-healthcare delivery. African Journal of Health Professions Education.

[22] National Institute for Communicable Diseases (NICD) VACCINE INFORMATION FOR PARENTS & CAREGIVERS.

[23] Breakwell L, Tevi-Benissan C, Childs L, Mihigo R, Tohme R (2017). The status of hepatitis B control in the African region. Pan Afr Med Journal.

[24] Massyn N, Pillay Y, Padarath A (2019). District health barometer 2017/18.

[25] Moonsamy S, Prabdial-Singh N, Motaze NV, Hardie D, Cohen C, Suchard M (2018). Laboratory-based Hepatitis B surveillance in South Africa 2018.

[26] National Institute for Communicable Diseases (NICD) (2023). Notifiable Medical Conditions Overview.

[27] World Health Organization (WHO) (2016). Global health sector strategy on viral hepatitis 2016-2021. Towards ending viral hepatitis. World Health Organization.

[28] Ward JW, Hinman AR (2019). What Is Needed to Eliminate Hepatitis B Virus and Hepatitis C Virus as Global Health Threats. Gastroenterology.

[29] Liu WC, Liu QY (2014). Molecular mechanisms of gender disparity in hepatitis B virus-associated hepatocellular carcinoma. World journal of gastroenterology.

[30] Yang F, Yin Y, Wang F, Zhang L, Wang Y, Sun S (2010). An altered pattern of liver apolipoprotein A-I isoforms is implicated in male chronic hepatitis B progression. Journal of proteome research.

[31] Verywell Health An Overview of Hepatitis B: Men are at greater risk for the disease.

[32] Price H, Dunn D, Zachary T, Vudriko T, Chirara M, Kityo C (2017). Hepatitis B serological markers and plasma DNA concentrations. AIDS (London. England).

[33] Scheibe A, Young K, Moses L, Basson RL, Versfeld A, Spearman CW (2019). Understanding hepatitis B, hepatitis C and HIV among people who inject drugs in South Africa: findings from a three-city cross-sectional survey. Harm reduction journal.

[34] Ruggieri A, Gagliardi MC, Anticoli S (2018). Sex-Dependent Outcome of Hepatitis B and C Viruses Infections: Synergy of Sex Hormones and Immune Responses?. Frontiers in immunology.

[35] Statistics South Africa (Stats SA) (2018). Mid-year population estimates 2018.

[36] Burnett RJ, Kramvis A, Dochez C, Meheus A (2012). An update after 16 years of hepatitis B vaccination in South Africa. Vaccine.

[37] MacLachlan JH, Cowie BC (2015). Hepatitis B virus epidemiology. Cold Spring Harbor perspectives in medicine.

[38] World Health Organization Hepatitis B Key Facts.

[39] WITS RHI Universal Test and Treat policy and adolescents in South Africa.

[40] Inoue T, Tanaka Y (2016). Hepatitis B virus and its sexually transmitted infection-an update. Microbial cell (Graz, Austria).

[41] Statistics South Africa (SSA): Stats SA (2018). Mid-year population estimates 2018.

[42] Woldesenbet S, Kufa T, Lombard C, Manda S, Ayalew K, Cheyip M (2018). The 2017 National Antenatal Sentinel HIV Survey. South Africa. National Department of Health.

[43] Okonkwo UC, Okpara H, Otu A, Ameh S, Ogarekpe Y, Osim H (2017). Prevalence of hepatitis B, hepatitis C and human immunodeficiency viruses, and evaluation of risk factors for transmission: Report of a population screening in Nigeria. S Afr Med J.

[44] London WT, Drew JS (1977). Sex differences in response to hepatitis B infection among patients receiving chronic dialysis treatment. Proceedings of the National Academy of Sciences.

[45] Khan F, Shams S, Qureshi ID, Israr M, Khan H, Sarwar MT (2011). Hepatitis B virus infection among different sex and age groups in Pakistani Punjab. Virology journal.

[46] Hecht R, Hiebert L, Spearman WC, Sonderup MW, Guthrie T, Hallett TB (2018). The investment case for hepatitis B and C in South Africa: adaptation and innovation in policy analysis for disease program scale-up. Health policy and planning.

